# Effects of Probiotic Strains on Disease Activity and Enteric Permeability in Psoriatic Arthritis–A Pilot Open-Label Study

**DOI:** 10.3390/nu12082337

**Published:** 2020-08-05

**Authors:** Andreas Haidmayer, Philipp Bosch, Angelika Lackner, Monica D’Orazio, Johannes Fessler, Martin H Stradner

**Affiliations:** 1 Department of Rheumatology and Immunology, Medical University of Graz, 8036 Graz, Austria; a.haidmayer@medunigraz.at (A.H.); philipp.bosch@medunigraz.at (P.B.); angelika.lackner@medunigraz.at (A.L.); monica.dorazio@medunigraz.at (M.D.); martin.stradner@medunigraz.at (M.H.S.); 2 Center for Biomarker Research in Medicine (CBmed), 8010 Graz, Austria

**Keywords:** psoriasis arthritis, zonulin, Th17 cells, probiotics, spondyloarthritis

## Abstract

(1) Background: Psoriatic Arthritis (PsA) is a painful disease of the joints and spine. Recent reports observed distinct enteric dysbiosis in PsA; intake of probiotic strains is considered to ameliorate enteric dysbiosis. If probiotics are effective in PsA is elusive. (2) Methods: In this pilot open-label study we enrolled 10 PsA patients with low to medium disease activity who received probiotics for 12 weeks. Analysis of faecal zonulin, α1-antitrypsin and calprotectin, as well as peripheral immune phenotyping was performed at baseline, after 12 weeks and 12 weeks after termination of probiotic intake. (3) Results: All patients showed increased levels of the enteric permeability marker zonulin which correlated with the frequency of peripheral Th17 cells. Calprotectin, a marker for intestinal inflammation was elevated in 6 out of 10 patients. Probiotic intake resulted in a reduction of disease activity and gut permeability. These effects, however, were not sustained beyond termination of probiotic intake. (4) Conclusions: PsA patients suffer from enhanced enteric permeability and inflammation. Probiotics may ameliorate disease activity in PsA by targeting these alterations.

## 1. Introduction

Psoriatic Arthritis (PsA) is the most prevalent coexisting condition of psoriasis affecting one third of the patients. The disease is characterized by painful and often disabling inflammation of the joints, entheses and spine [1]. PsA belongs to the family of spondyloarthritis (SpA) sharing features with ankylosing spondylitis, reactive arthritis and enteropathic arthritis. Pathophysiologically, SpA has been linked to bacterial triggers; the HLA-B27 transgenic rat model of SpA does not develop disease in a germ free environment [2]. Similarly, SKG mice develop a PsA-like disease depending on the presence of bacterial colonization or injection of β-glycan, a polysaccharide found in bacterial and fungal cell walls. In this model CD4^+^ T-cell immune responses and interleukin-17 (IL-17) are involved in the development of arthritis [3,4]. Intriguingly, IL-17 secreting CD4^+^ T-cells (Th17) physiologically fight bacterial and fungal infection. Moreover, blocking IL-17 by monoclonal antibodies is an effective therapy for psoriasis and PsA [5]. Intestinal Th17 cells can be suppressed or induced depending on the composition of the gut microbiota and the metabolites they produce. Short chains fatty acids (SCFA) released by Faecalibacterium, Ruminococcus and Akkermansia species suppress IL-17 production and induce anti-inflammatory regulatory T-cells (Treg) [6]. Frequencies of Ruminococcus and Akkermansia species were reported to be reduced in the Ileum of PsA patients compared to psoriasis patients without joint involvement and healthy controls [7]. Enteric dysbiosis was also observed in ankylosing spondylitis and enteropathic arthritis [8,9]. Histologic analyses of biopsies obtained from the ileum of these patients revealed downregulation of epithelial tight junctions associated with bacterial invasion into the mucosa and increased expression of zonulin, a protein modulating enteric tight junctions and thus intestinal permeability.

Enteric dysbiosis can be ameliorated by dietary measures, stool transplantation, or intake of probiotic strains [10,11]. Oral administration of probiotic Bifidobacterium and Lactobacillus strains significantly improved psoriatic skin lesions compared to placebo in a randomized controlled trial [12]. Based on this data we hypothesize that probiotic strains may amend dysbiosis in PsA leading to improvement of the disease activity. Here we present evidence for enteric inflammation and mucosal permeability. In an open-label pilot study, administration of probiotic strains over a 12-week period improved signs of inflammation and enteric permeability and led to a significant decrease in disease activity.

## 2. Materials and Methods

### 2.1. Study Population

This open-label pilot-study was performed from December 2018 to December 2019. The ethics board of the Medical University of Graz approved the study protocol (30–166 ex 17/18). Patients visiting the rheumatology outpatient clinic were asked to participate in the study in case they met the inclusion criteria: (1) diagnosis of PsA diagnosed by a rheumatologist and satisfied CASPAR criteria, (2) low or moderate disease activity, (3) age above 18 years, (4) stable immunomodulatory therapy over 3 months. Exclusion criteria were: (1) history of bariatric surgery, (2) use of other probiotic products, (3) antibiotic therapy within the last month before inclusion, (4) inflammatory bowel disease, (5) acute myocardial infarction or decompensated heart failure within the last 3 months, (6) stroke within the last 3 months, (7) history of malignancy or any other condition or circumstance, which would affect the patient’s ability to participate in the protocol, (8) pregnancy. Criteria for withdrawal from the study were serious adverse events, changes in the background medication and other protocol deviation. Informed consent was obtained from all individuals prior to study initiation.

### 2.2. Procedure

From baseline to week 12 all patients received 3 g of probiotic powder dissolved in tap water once daily containing corn starch, maltodextrin, fructo-oligosaccharide P6, inulin P2, vegetable protein and nine bacterial strains of *Lactobacillus* and *Bifidobacterium* with at least 7.5 billion organisms per portion (OMNi-BiOTiC^®^ STRESS Repair, Institut Allergosan, Graz, Austria; details of composition see Suppl. Table S1). All strains confer with safety certificates recognized by the European Food Safety Authority. Medical history, clinical examinations, evaluation of disease activity by modified psoriatic arthritis disease activity score (mPASDAS) [13,14], blood and stool samples were obtained at baseline, after 12 and 24 weeks. Telephone interviews were performed after 6 weeks to ensure compliance and identify potential adverse events. The primary endpoint of the study was disease activity in order to calculate the sample size for a future randomized controlled trial. The secondary endpoints were exploratory and included levels of fecal calprotectin, zonulin and α1-antitrypsin, plasma sCD14 and LPS-binding protein, and analysis of T and B cell subsets in peripheral blood.

### 2.3. ELISA

Feces samples were collected and stored at −80 °C until analysis. Calprotectin, α1-antitrypsin and zonulin concentrations were determined by using enzyme-linked immunosorbent assay (ELISA) kits (Immundiagnostik AG, Bensheim, Germany, K 6927 (calprotectin); K 6750 (α1-antitrypsin); K 5600 (zonulin)). All assays were carried out in accordance with the manufacturer’s manual. Samples were read on a microplate reader (Dynex, Chantilly, USA, DSX) at 450 nm. Standard curves and calculations are based on 4-parameter logistic regression (4PL). All measurements were performed in duplicates.

Plasma samples were stored at −80 °C until analysis. sCD14 and LBP concentrations were determined by using ELISA kits (R&D systems, Abington, UK and Hycult biotech, Uden, Netherlands). All assays were carried out in accordance with the manufacturer’s manual. All measurements were performed in duplicates.

### 2.4. Flow cytometry

FACS analysis of T and B-cell subsets was performed as previously described [15]. In brief, erythrocytes were lysed and leukocytes were incubated with antibodies against CD3 (Becton Dickinson, San Diego, USA), CD4, CD8, CD28, CD31, CD45, CD45RO (all Beckman Coulter, Brea; USA), CD19, CD197/CCR7, CD127, CD183/CXCR3, CD196/CCR6, CD194/CCR4 (all Miltenyi biotech, Bergisch Gladbach, Germany) and CD38 and CD45RA (Becton Dickinson) for T-cell phenotyping and CD19, CD21, CD24, CD27, CD38 IgD and IgM (all Miltenyi biotech) for B-cell phenotyping. For intracellular staining of Ki-67 (Becton Dickinson), cells were permeabilized with IntraPrep Reagents (Beckman Coulter; Brea, USA) and measured within 2 h. These analyses were performed on a FACS Canto II (Becton Dickinson) using FACS Diva and FlowJo software (Becton Dickinson).

### 2.5. Statistics

All statistical analyses were performed using SPSS, v23.0 (Chicago, IL, USA). All data are presented as median (range) and we used the Mann-Whitney-U for independent or Wilcoxon test for dependent comparisons. Correlation between variables was determined by the Spearman’s rank correlation coefficient.

## 3. Results

Ten patients were enrolled in the study. Clinical characteristics are described in Table 1. Four patients had low and six patients had moderate disease activity according to their mPASDAS score. Three patients did not complete the study: One patient refused to continue probiotic intake after 20 days due to flatulence. In two patients, the background medication was changed during the first 12 weeks, resulting in a protocol deviation and exclusion. Therefore, data of these patients were analyzed only at baseline. No severe adverse events occurred throughout the study.

### 3.1. Probiotics Intake Decreases Disease Activity in PsA Patients

An expanding body of evidence from animal and human studies has shown therapeutic and preventive potency of probiotics intake on a number of disorders including inflammatory and autoimmune diseases, as well as infections and cancers [16,17,18].

**Figure 1:**
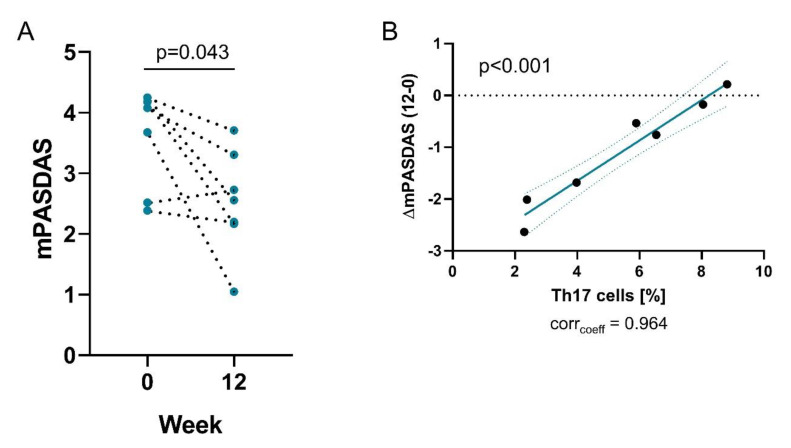
Probiotics treatment decreases disease activity. Graphs show (**A**) mPASDAS score at baseline (0) and week 12 of treatment with probiotics; (**B**) Correlation of the change of PASDAS from baseline to week 12 with the frequencies of Th17 cells (right).

Analyzing the primary end-point of this study we observed that disease activity of PsA patients decreased significantly after probiotic intake (Figure 1A and Suppl. Table S2). Three patients with medium disease activity changed to low disease activity. These data will allow calculation of the sample size for a randomized placebo-controlled study. Interestingly, the change in disease activity was correlated with the frequency of peripheral Th17 cells at baseline (Figure 1B), suggesting that low levels of Th17 cells are associated with a better response to probiotic intake. The frequency of total or proliferating peripheral Th17 cells as well as Th1, Th2 and Treg cells were not affected by probiotic treatment.

### 3.2. Intestinal Permeability is Enhanced in PsA Patients

**Figure 2:**
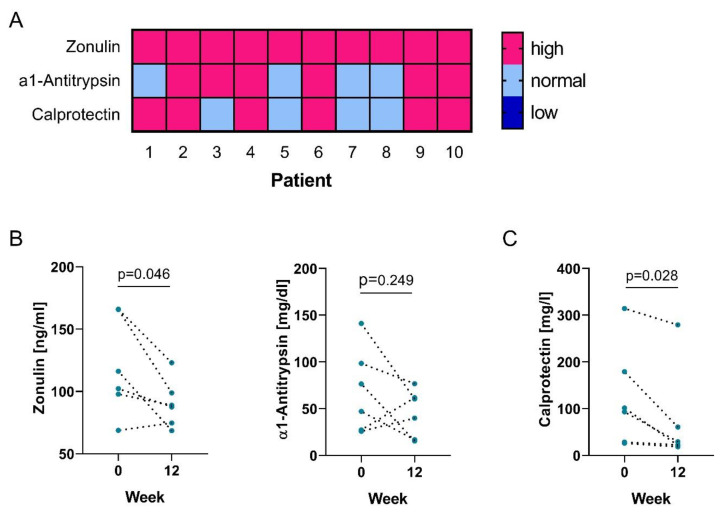
Intestinal permeability is increased in PsA patients and ameliorates with probiotics intake. (**A**) Heatmap showing too low (dark blue), normal (light blue) and too high (pink) levels of markers of gut permeability (zonulin and α1-antitrypsin) and intestinal inflammation (calprotectin) in PsA patients; Graphs show (**B**) fecal concentrations of zonulin (left) and α1-antitrypsin (right); (**C**) fecal concentrations of calprotectin at baseline (0) and week 12 of treatment with probiotics.

Opening of intercellular tight junctions in the gastrointestinal tract and thus increased intestinal permeability can act as a trigger for diseases such as Crohn’s disease or IBD. [19] Intestinal permeability, moreover, is likely influenced by the gut microbiome [20]. Given the marked microbial dysbiosis reported in PsA patients [7], we wanted to determine if gut permeability was altered in our patient cohort. Therefore, we measured fecal markers zonulin and α1-antitrypsin. Levels of fecal zonulin exceeded the upper of normal (50 ng/mL) in all patients, whereas abnormally high levels of α1-antitrypsin were found in 60% of the patients (Figure 2A).

In order to determine if intestinal permeability is associated with gut inflammation, we also tested fecal specimen for calprotectin levels, a protein that is released from neutrophils upon activation. Again, we observed that 60% of the patients showed elevated levels of fecal calprotectin (Figure 2A). Interestingly, 5 out of these 6 patients also had elevated α1-antitrypsin. Together, these data suggest that PsA patients suffer from increased enteric permeability and inflammation.

Bifidobacterium and Lactobacillus strains were previously reported to be beneficial for intestinal homeostasis of the microbiome [21,22,23]. Therefore, we evaluated the potential of probiotics to ameliorate the increased intestinal permeability observed in our patient cohort.

**Figure 3:**
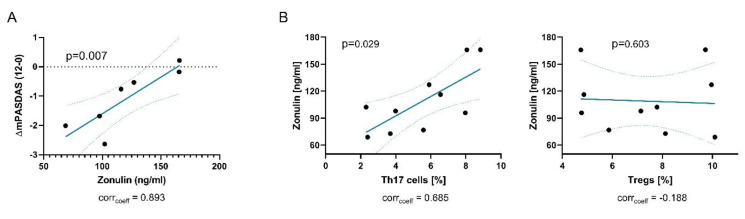
Intestinal permeability is a predictor for probiotics effect. Graphs show (**A**) correlation of the change of PASDAS from baseline to week 12 with zonulin; (**B**) correlations of zonulin with the frequency of Th17 cells (left) and Tregs (right).

We noticed a significant decrease of fecal zonulin in our patient cohort following probiotics intake (Figure 2B). α1-antitrypsin levels on the other hand remained unchanged overall, but patients with high levels showed a decrease following treatment suggesting a positive effect of the probiotics intake on gut permeability. Furthermore, we observed a significant decrease of fecal calprotectin levels and a reduction of patients with abnormal high calprotectin levels to 33%, indicating a reduction in gut inflammation (Figure 2C). Of note, the change in disease activity was correlated with baseline levels of fecal zonulin (Figure 3A), indicating that low levels of zonulin are associated with a better response to probiotic intake.

### 3.3. Intestinal Permeability is Linked to a Th17-Related Immune Response

Next, we wanted to investigate if the observed alterations are connected to immunological parameters in the periphery. Of note, levels of fecal zonulin correlated with the frequency of peripheral Th17 cells, but not Th1, Th2 or regulatory T-cells (Tregs) (Figure 3B), suggesting that increased gut permeability drives peripheral Th17 cell immune responses or vice versa. Proliferating Ki67^+^ Th17 cells were also correlated with mPASDAS disease activity score (corr_coeff_ = 0.745, p=0.013) and a trend of correlation was observed with fecal calprotectin levels as a marker of intestinal inflammation (corr_coeff_ = 0.576, *p* = 0.082).

Furthermore, we were interested in LPS-response associated proteins lipopolysaccharide binding protein (LBP) and soluble CD14 (sCD14). Plasma concentrations of both did not correlate with disease activity, markers of gut permeability or gut inflammation. Plasma LBP and sCD14 levels, however, significantly correlated with the frequency of peripheral Th1 cells (LBP: corr_coeff_ = 0.697, *p* = 0.025; sCD14: corr_coeff_ = 0.636, *p* = 0.048). In addition, concentrations of LBP and sCD14 remained unchanged following probiotics intake, leading to the suggestion that LBP and sCD14 are independent of alterations of gut permeability and inflammation.

### 3.4. Effects of Probiotics are not Long-Lasting

After 12 weeks probiotic intake was terminated. We performed a follow-up visit after another 12 weeks to investigate for long lasting treatment effects.

**Figure 4:**
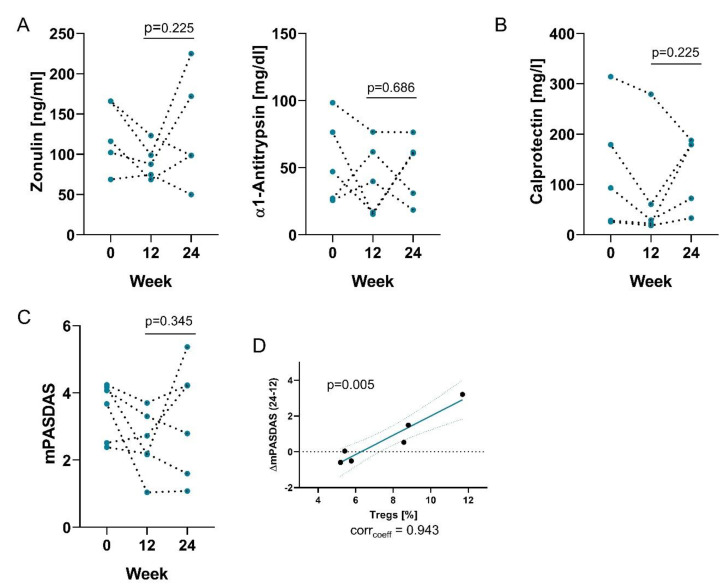
Effects of probiotics are not long-lasting. Graphs show (**A**) fecal concentrations of zonulin (left) and α1-antitrypsin (right); (**B**) fecal concentrations of calprotectin; (**C**) mPASDAS score at baseline, week 12 and 24 following the termination of probiotics treatment; (**D**) Correlations of the change of PASDAS from week 12 to 24 with the frequency of Tregs.

Fecal zonulin levels increased in response to termination of probiotic treatment in 3 of 5 of PsA patients (Figure 4A) suggesting that the effect of probiotics intake on gut permeability may not be long lasting. Analyzing the gut inflammation marker calprotectin, we observed that 4 of 5 PsA patients showed elevated levels compared to week 12 (Figure 4B). 5 of 6 of PsA patients had abnormal high levels of fecal calprotectin at week 24 in comparison to 2 of 6 patients at week 12.

Consequently, 4 of 6 PsA patients showed aggravated disease activity according to their mPASDAS score (Figure 4C) leading to a change in disease category from low to medium for two patients. Interestingly, the change in mPASDAS was positively correlated with the frequency of peripheral Tregs at week 12, showing that patients that worsened had higher levels of regulatory cells (Figure 4D).

## 4. Discussion

In this pilot study, we found evidence of elevated gut permeability and inflammation in PsA patients. Intake of probiotic strains led to a decrease in intestinal permeability and, more importantly, to amelioration of disease activity. These effects, however, were not long-lasting following termination of the treatment. These preliminary data demonstrate the need for an extended clinical trial to assess the potency of probiotics intake for the modulation of PsA disease activity.

Accumulating evidence has linked the intestinal microbiota to inflammation. Intestinal dysbiosis is considered a potential risk factor for chronic inflammation, even at distant sites like the joint [24]. Already in 1996, seminal work evaluated the role of the gut microbiome in the development of arthritis as well as colitis in the HLA-B27 transgenic rat model [2]. Since then, a great number of studies have contributed to establish a link of gut microbial dysbiosis and an excessive immune response in the intestinal lamina propria, subsequently leading to systemic inflammation and finally joint disease. Recently, studies reported PsA-like gut and joint pathology does not develop under germ-free conditions shown in SKG and TNF^ΔARE/+^ mice [4,25]. Moreover, a state of microbial dysbiosis was discovered in PsA patients [7,26]. Scher et al. reported a distinct reduction in Ruminococcus and *A. municiphila* among others in 16 PsA patients [7]. Interestingly, similar results were found in patients suffering from IBD suggesting a shared mechanism [27]. Several Ruminococcus species as well as *A. municiphila* are mucus-degrading gut symbionts that convert mucin into short-chain fatty acids (SCFAs). In an elegant study, SCFAs were reported to be potent immunomodulators by suppressing Th17 cells and autoimmunity [28]. Interestingly, we observed that the occurrence of peripheral Th17 cells correlated significantly with gut permeability marker zonulin, which is likely dependent on microbiome composition. IL-17 producing cells are thought to be major driver of PsA pathology and anti-IL-17 therapy is a successful treatment option [5]. Subsequently, it seems possible that bacterial antigens or cross-reactive T-cells, activated in the intestine home the joint. The precise immunological mechanisms linking gut and joint inflammation, however, are not completely understood.

We found markedly increased fecal zonulin in all our patients. In line with these findings, increased zonulin expression was also observed in other diseases of the SpA spectrum such as ankylosing spondylitis and enteropathic arthritis [8,9]. Interestingly, ileal biopsies of patients with enteropathic arthritis showed more pronounced bacterial infiltration, epithelial damage, and mucosal permeability than those of patients with inflammatory bowel disease alone. Thus, increased enteric permeability may promote the development of SpA. In our study, intake of probiotics significantly reduced zonulin levels. Similar results were observed in patients suffering from Alzheimer’s disease [29]. In our patients, clinical response to probiotics inversely correlated with zonulin levels and frequency of Th17 cells at baseline. We hypothesize that a short course of probiotics may likely restore barrier function in PsA patients with mildly increased enteric permeability while patients with more pronounced changes may require a longer probiotic treatment than just 12 weeks.

This study has limitations: It was an open-label single arm pilot study on a small number of PsA patients thus limiting clinical conclusions. A larger adequately powered randomized controlled study based on the results of this study is underway to assess clinical efficacy of probiotics in PsA. Also, we performed T- and B-cell phenotyping only from whole blood. Alterations of frequency and function of intestinal and synovial immune cells would probably increase the significance of the findings.

Taken together this study shows elevated gut permeability in PsA patients. Probiotics intake led to amelioration of enteric permeability and disease activity suggesting that probiotics may be beneficial for PsA patients in addition to conventional therapy.

## Figures and Tables

**Table 1:** Patient characteristics.

	*n* = 10
Age at inclusion, years, median (range)	58 (51–72)
Disease duration, years, median (range)	11 (2–33)
Female sex, *n*	7
Current smoker, *n*	3
Anti-TNF, *n*	3
Anti-IL-17, *n* (%)	2
Methotrexate, *n* (%)	2
NSAID, *n* (%)	5
PASI, median (range)	0.3 (0–4.2)
mPASDAS, median (range)	3.9 (2.51–4.53)

NSAID, non-steroidal anti-inflammatory drug; PASI, psoriasis area and severity index; mPASDAS, modified psoriatic arthritis disease activity score.

## Supplementary Materials

The following are available online at https://www.mdpi.com/2072-6643/12/8/2337/s1, Table S1: Bacterial composition of OMNi-BiOTiC® STRESS Repair, Table S2: Data of mPASDAS score elements at baseline as well as week 12 and 24.
