# Supplementary material for: Effects of Probiotic Strains on Disease Activity and Enteric Permeability in Psoriatic Arthritis–A Pilot Open-Label Study
Source: Nutrients. 2020 Aug 5;12(8):2337. doi: 10.3390/nu12082337 (PMC7468965; doi:10.3390/nu12082337)
Supplement: Supplementary file 1 [file nutrients-12-02337-s001.pdf]

Supplementary Table *1*. Bacterial composition of OMNi-BiOTiC® STRESS Repair:

Bifidobacterium bifidum W23
Bifidobacterium lactis W51
Bifidobacterium lactis W52
Lactobacillus acidophilus W22
Lactobacillus casei W56
Lactococcus lactis W19
Lactobacillus paracasei W20
Lactobacillus plantarum W62
Lactobacillus salivarius W24

Supplementary Table 2. Data of mPASDAS score elements at baseline as well as week 12 and 24*:*

parameter	baseline	week 12	week 24
CRP (mg/L)	2.7 (0.7-8.4)	2.1 (0.9-12.2)	1.9 (0.6-9.6)
Dactylitis count	0 (0-0)	0 (0-0)	0 (0-0)
EGA	30 (0-35)	0 (0-5)	0 (0-20)
HAQ	1 (0-1.38)	1.19 (0-1.88)	0.75 (0-1.75)
Leeds Enthesitis Count	0 (0-1)	0 (0-0)	0 (0-0)
PGA	35 (4-50)	45 (30-50)	30 (5-60)
SJC	0 (0-3)	0 (0-0)	0 (0-1)
TJC	7 (1-21)	3 (0-18)	1 (0-12)

Data are presented as median (range). CRP, C-reactive protein; EGA, evaluator's global assessment (range 0-100mm); HAQ, health assessment questionnaire (range 0-3); PGA, patient's global assessment (range 0-100mm); SJC, swollen joint count (range 0-66); TJC, tender joint count (range 0-68)
